# Traditional Chinese Medicine Compound Preparations Are Associated with Low Disease-Related Complication Rates in Patients with Rheumatoid Arthritis: A Retrospective Cohort Study of 11,074 Patients

**DOI:** 10.1155/2023/1019290

**Published:** 2023-02-23

**Authors:** Yanyan Fang, Jian Liu, Ling Xin, Xiaolu Chen, Xiang Ding, Qi Han, Mingyu He, Xu Li, Yanqiu Sun, Fanfan Wang, Jie Wang, Xin Wang, Jianting Wen, Xianheng Zhang, Qin Zhou, Junru Zhang

**Affiliations:** ^1^The First Affiliated Hospital of Anhui University of Chinese Medicine, Hefei, Anhui 230038, China; ^2^Anhui Province Key Laboratory of Modern Chinese Medicine Department of Internal Medicine Application Foundation Research and Development, Hefei, Anhui 230038, China

## Abstract

**Objective:**

To evaluate whether traditional Chinese medicine compound preparations (TCMCPs) are associated with rheumatoid arthritis- (RA-) related complications (including readmission, Sjogren's syndrome, surgical treatment, and all-cause death) in patients with RA.

**Methods:**

Clinical outcome data were retrospectively collected from patients with RA discharged from the Department of Rheumatology and Immunology of the First Affiliated Hospital of Anhui University of Chinese Medicine from January 2009 to June 2021. The propensity score matching method was used to match baseline data. Multivariate analysis was conducted to analyze sex, age, the incidence of hypertension, diabetes, and hyperlipidemia and identify the risk of readmission, Sjogren's syndrome, surgical treatment, and all-cause death. Users of TCMCP and nonusers of TCMCP were defined as the TCMCP and non-TCMCP groups, respectively.

**Results:**

A total of 11,074 patients with RA were included in the study. The median follow-up time was 54.85 months. After propensity score matching, the baseline data of TCMCP users corresponded with those of non-TCMCP users, with 3517 cases in each group. Retrospective analysis revealed that TCMCP significantly reduced clinical, immune, and inflammatory indices in patients with RA, and these indices were highly correlated. Notably, the composite endpoint prognosis for treatment failure in TCMCP users was better than that in non-TCMCP users (HR = 0.75 (0.71-0.80)). The risk of RA-related complications in TCMCP users with high-exposure intensity (HR = 0.669 (0.650-0.751)) and medium-exposure intensity (HR = 0.796 (0.691-0.918)) was significantly lower than those in non-TCMCP users. An increase in exposure intensity was associated with a concomitant decrease in the risk of RA-related complications.

**Conclusion:**

The use of TCMCPs, as well as long-term exposure to TCMCPs, may lower RA-related complications, including readmission, Sjogren's syndrome, surgical treatment, and all-cause death, in patients with RA.

## 1. Introduction

Rheumatoid arthritis (RA) is a chronic inflammatory disease that mainly causes gradual joint damage and affects other body systems [1, 2]. The worldwide incidence rate of RA is approximately 1%, and although this condition affects people of all ages, it is more prevalent in women than in men [3, 4]. Currently, the etiology of RA is unclear, which poses a challenge to the effective treatment of RA and increases rehospitalization rates [5]. Although synovitis is a primary pathological marker of RA, many extra-articular manifestations may occur because of RA's complex, chronic, inflammatory, and autoimmune characteristics [6–8]. Extra-articular manifestations and complications are common in RA, contributing to higher incidence rates and premature mortality [6]. A hallmark clinical feature of RA is the symmetrical polyarthritis that manifests as redness and pain in the joints, especially smaller joints, and long-term morning stiffness [9, 10], with the potential to progress to serious joint injury and disability [11]. Progressive and severe joint injury, chronic pain, loss of function, and insufficient response to treatment regimens are indications for final joint replacement surgery [12]. Cohort studies based on national data from several countries have shown that RA is associated with high mortality [13, 14]. Therefore, readmission, extra-articular manifestations, surgical treatment, and all-cause death are considered potential RA-related complications.

Modern pharmacological treatments for RA mainly include nonsteroidal anti-inflammatory drugs, glucocorticoids, conventional disease-modifying antirheumatic drugs (cDMARDs), and biologic DMARDs that are used to alleviate chronic pain in patients by reducing the local inflammatory response [15]. However, RA treatment is complex and requires the combined application of multiple drugs, some of which have significant side effects and high treatment costs, resulting in poor patient compliance. Traditional Chinese medicine (TCM) might have many therapeutic advantages for RA [16–18]. Xin'an Jianpi Tongbi prescription, including Xinfeng capsule (XFC), Huangqin Qingre Chubi capsule (HQC), and Wuwei Wentong Chubi capsule (WWT), is a routinely used TCM compound preparation (TCMCP), which contains *Astragalus membranaceus*, *Semen coicis*, *Tripterygium wilfordii*, *Scolopendra* spp., *Scutellaria baicalensis*, *Gardenia jasminoides*, *Prunus persica*, *Clematis chinensis*, *Poria cocos*, *Epimedium brevicornu*, *Cinnamomum cassia*, *Curcumae Longae*, and other drugs. Many studies have shown that this TCMCP has high efficacy against RA [18–20]. A randomized, double-blind, multicenter, and placebo-controlled trial showed high efficacy and safety of XFC in the treatment of patients with RA [21, 22]. Animal experiments have demonstrated that HQC improves the baseline severity of arthritis in a collagen-induced arthritis mouse model [23, 24]. WWT has also been reported to have a good pharmacological effect on RA [25]. However, although the TCMCPs have favorable therapeutic effects on RA, their specific effect on the incidence of RA-related complications is still unclear.

In this study, we retrospectively analyzed the effect of Xin'an Jianpi Tongbi prescription on immune inflammation in RA and the risk of four RA-related complications, including readmission, Sjogren's syndrome, surgical treatment, and all-cause death.

## 2. Methods

### 2.1. Study Cohort (Figure 1)

Clinical data of discharged patients with RA from the Department of Rheumatology and Immunology of the First Affiliated Hospital of Anhui University of Chinese Medicine were retrospectively collected from January 2009 to June 2021. The diagnostic criteria for RA by the American College of Rheumatology were adopted in this study [26]. Telephonic follow-up time was calculated from the time of discharge until February 28, 2022. Based on the history of TCMCP usage, the risk of RA-related complications, including readmissions, Sjogren's syndrome, surgical treatments, and all-cause death, was evaluated. This study was approved by the Medical Ethics Committee of the First Affiliated Hospital of Anhui University of Chinese Medicine (approval number: 2022MCZQ01).

### 2.2. Data Collection

Demographic information, including age and sex; clinical data including baseline complications, baseline cDMARD, and corticosteroid treatment; and data on TCMCPs were collected and evaluated retrospectively.

### 2.3. Treatment

In the First Affiliated Hospital of Anhui University of Chinese Medicine, the basic drugs for treating RA consist of cDMARDs (including methotrexate, leflunomide, sulfasalazine, and hydroxychloroquine sulfate), nonsteroidal anti-inflammatory drugs (including celecoxib, meloxicam, and lornoxicam), and glucocorticoids (methylprednisolone). It should be noted that TCM is a commonly used treatment means in TCM hospitals. We gradually withdrew the use of biologics by increasing the use of TCM.

### 2.4. Inflammatory and Immune Indices

Inflammatory and immune indices, including erythrocyte sedimentation rate (ESR), C-reactive protein (CRP), anti-cyclic citrullinated peptide (anti-CCP), rheumatoid factor (RF), immunoglobulin A (IgA), immunoglobulin G (IgG), immunoglobulin M (IgM), complement component 3 (C3), and complement component 4 (C4) levels, were evaluated after TCMCP treatment.

### 2.5. Research Definition

#### 2.5.1. Xin'an Jianpi Tongbi Prescription

Xin'an Jianpi Tongbi prescription is a compound preparation of TCM based on the Xin'an medical theory. It contains Xinfeng capsule [22] (Z20050062 from Wanyao Pharmaceutical Co., Ltd., patent number: ZL 2013 1 0011369.8), Huangqin Qingre Chubi capsule [24] (Z20200001 from Wanyao Pharmaceutical Co., Ltd., patent number: ZL 2011 1 0095718.X), and Wuwei Wentong Chubi capsule [25] (patent number: ZL 2020 10714863.0). The Xinfeng capsule is composed of *Astragalus membranaceus*, *Semen coicis*, *Tripterygium wilfordii*, and *Scolopendra* spp. These four medicinal materials were extracted by refluxing twice with 75% ethanol. In the first step, ten times the amount of ethanol was added for extraction for 2 h, after which eight times the amount of ethanol was added and allowed to extract for 1.5 h. The drug residues were boiled with eight times the amount of water and extracted for 1.5 h. This was then filtered and allowed to stand. The supernatant was collected and combined with the alcohol extract under pressure to concentrate, and the paste was collected. The sample was dried, crushed, mixed with dextrin, and granulated with ethanol. This was followed by drying, whole granulating, sterilizing, filling, and outsourcing. The Huangqin Qingre Chubi capsule is composed of *Scutellaria baicalensis*, *Prunus persica*, *Gardenia jasminoides*, *Semen coicis*, and *Clematis chinensis*. These five medicinal materials were decocted and extracted three times as follows: ten times the amount of water was added for the first time and extracted for 1.5 h; eight times the amount of water was added for the second and third times and extracted for 1 h. The mixture was strained and allowed to stand. Then, the supernatant was absorbed and concentrated under pressure, and the paste was collected; this was then vacuum-dried, the dry extract was crushed, and dextrin was added. Ethanol was used to soften the materials, which were screened, granulated, dried, whole-grained, and filled into capsules. The Wuwei Wentong Chubi capsule is composed of *Poria cocos*, *Epimedium brevicornu*, *Cinnamomum cassia*, *Curcumae Longae*, and *Scutellaria baicalensis*. These five medicinal materials were decocted and extracted three times as follows: ten times the amount of water was added for the first time and extracted for 1.5 h; eight times the amount of water was added for the second and third times and extracted for 1 h. This mixture was strained and allowed to settle. Then, the supernatant was absorbed and concentrated under reduced pressure, and the paste was collected. This was then vacuum-dried, the dry extract was crushed, and dextrin was added. Ethanol was used to soften the material, which was then sieved using no. 12 mesh, granulated, dried, whole-grained, filled into capsules, and outsourced. All capsules were produced by the preparation center of the First Affiliated Hospital of Anhui University of Chinese medicine, and the variation range of each capsule was ±10%.

#### 2.5.2. RA-Related Complications

Readmission refers to RA patients who have been hospitalized twice or more. Sjogren's syndrome refers to an RA-related complication with a frequency of 10.41% (1022/9813). Surgical treatment refers to RA patients with severe joint deformities requiring surgical treatment. All-cause death refers to death caused by long-term RA distress.

#### 2.5.3. Classification of Quantitative Variables

The usage of TCMCPs was defined as “1,” and nonusage was defined as “0.” After TCMCP treatment, a decrease in ESR, CRP, IgA, IgG, IgM, C3, C4, RF, and anti-CCP levels was recorded as “1,” whereas an increase or no change in the level was recorded as “0.” The decrease in inflammatory and immune index values indicated the effectiveness of TCMCP treatment.

#### 2.5.4. Exposure Intensity

According to exposure intensity, patients who received TCMCPs for less than 1 month, 1–3 months, 3–6 months, and ≥6 months after discharge were defined as the nonexposure, low-exposure, medium-exposure, and high-exposure groups, respectively.

### 2.6. Statistical Analysis

Continuous variables are reported as medians with interquartile ranges (IQR), whereas categorical variables are reported as frequencies and percentages. Categorical variables were compared using Fisher's exact test, whereas continuous variables were compared using the Wilcoxon signed-rank test. Univariate and multivariate COX proportional hazards models were developed to evaluate risk factors for the occurrence of endpoint events and are presented as hazard ratios (HR with 95% confidence intervals (CIs)). Univariate models contained a single predictor for calculating different baseline risks for each site. Multivariate models included age, sex, comorbidities at baseline, and TCMCP as model covariates. All analyses were performed using SPSS V.22 (Armonk, NY, USA) software. Differences were considered statistically significant when the *p* value was less than 0.05.

## 3. Results

### 3.1. Baseline Characteristics of TCMCP and Non-TCMCP Patients (Table 1)

The baseline data, including sex, age, hypertension, diabetes, hyperlipidemia, and cDMARD and corticosteroid treatment, of 9813 patients with RA who were successfully followed up were recorded. The median follow-up time was 54.85 months. Before propensity score matching, there were significant differences between TCMCP users and non-TCMCP users in terms of sex, age, hypertension, diabetes, cDMARDs, and corticosteroid treatment (*p* < 0.05). However, after matching, no significant difference was found between TCMCP users and non-TCMCP users in the same aspects (*p* > 0.05).

### 3.2. Changes in the RA-Related Inflammatory and Immune Indices after TCMCP Treatment (Table 2)

Hospitalization data of 3517 patients in the matched TCMCP group who received Xin'an Jianpi Tongbi prescription during hospitalization were collected and analyzed. Their posttreatment inflammatory and immune indices were lower than those before treatment (*p* < 0.05).

### 3.3. Association Analysis of TCMCPs with RA-Related Inflammatory and Immune Indices (Table 3)

We further analyzed the association between Xin'an Jianpi Tongbi prescription and RA-related inflammatory and immune indices. The results indicated that XFC was positively correlated with a decrease in CRP (*p* = 0.039, OR = 1.216), ESR (*p* = 0.003, OR = 1.298), and C4 (*p* = 0.028, OR = 1.258) levels. Similarly, HQC was positively correlated with a decrease in CRP (*p* < 0.001, OR = 1.641), ESR (*p* = 0.002, OR = 1.324), C4 (*p* = 0.024, OR = 1.272), IgG (*p* = 0.019, OR = 1.247), and IgA (*p* = 0.022, OR = 1.237) levels.

### 3.4. Kaplan-Meier Curves for a Composite Endpoint for Treatment Failure for TCMCP Users versus Non-TCMCP Users (Figure 2)

The results of the log-rank test showed that TCMCP users had better composite endpoint prognoses for treatment failure (HR = 0.75 (0.71-0.80), *p* < 0.001) than non-TCMCP users.

### 3.5. COX Regression Model for Analysis of Risk Factors for Four RA-Related Complications (Table 4) and Visualization of the Analysis Results (Figure 3)

Further, we used univariate and multivariate COX regression to analyze risk factors for the four RA-related complications, namely, readmission, Sjogren's syndrome, surgical treatment, and all-cause death. The results showed that TCMCPs reduced the risk of readmission, Sjogren's syndrome, surgical treatment, and risk of all-cause death by 20.7%, 32.6%, 29.0%, and 27.4%, respectively.

Advancing age increased the risk of Sjogren's syndrome, surgical treatment, and all-cause death by 60.5%, 94.7%, and 106.0%, respectively. The comorbidity hypertension increased the risk of readmission, Sjogren's syndrome, and all-cause death by 51.9%, 56.6%, and 50.6%, respectively. The male sex and the presence of comorbidity diabetes increased the risk of all-cause death by 51.7% and 59.6%, respectively. Hyperlipidemia had a 34.0% increased risk of readmission.

### 3.6. Risk of RA-Related Complications at Different Exposure Times (Table 5)

We found that the use of TCMCPs was associated with a lower risk of RA-related complications. In addition, the risk of RA-related complications varied according to the exposure time. Notably, the risk of RA-related complications in TCMCP users with high-exposure intensity (adjusted HR = 0.699, 95%CI = 0.650-0.751, *p* < 0.001) and medium-exposure intensity (adjusted HR = 0.796, 95%CI =0.691-0.918, *p* = 0.002) was significantly lower than that in non-TCMCP patients.

## 4. Discussion

In this population-based cohort study, a large amount of data on RA patients from the First Affiliated Hospital of Anhui University of Chinese Medicine were used to evaluate the effects of TCMCPs on clinical immunological and inflammatory indicators and RA-related complications. We found that RA patients treated with Xin'an Jianpi Tongbi Preparation not only exhibited lower immune and inflammatory indices than non-TCMCP users but also were associated with a low risk of RA-related complications.

TCM has a multicomponent, multitargeted synergistic anti-inflammatory and anti-immune effect. Previously, we found that TCMCPs significantly improved the RA-related immunological and inflammatory effects [16]. Modern pharmacological studies have also reported that Xin'an Jianpi Tongbi preparation drugs, i.e., *Astragalus membranaceus*, *Semen coicis*, *Tripterygium wilfordii*, *Scolopendra spp.*, *Scutellaria baicalensis*, *Gardenia jasminoides*, *Poria cocos*, *Epimedium brevicornu*, *Cinnamomum cassia*, and *Curcumae Longae*, can improve the RA-related immunological and inflammatory response. Among them, active agents in *Astragalus membranaceus* have been shown to improve RA-induced synovial and joint injury [27, 28]. *Semen coicis* extract, including polyphenols and polysaccharides, has immunological, antioxidant, and anti-inflammatory effects [29]. *Tripterygium wilfordii* lactone, the active ingredient of *Tripterygium wilfordii*[30, 31], inhibited cell growth and inflammatory response of RA-associated fibroblasts, such as synovial cells, by regulating the expression of the hsa-circ-0003353/microRNA-31-5p/cyclin-dependent kinase 1 axis [32]. *Scolopendra* spp. combined with TCM has shown significant clinical efficacy in patients with RA [33]. Baicalin had an anti-inflammatory effect in a collagen-induced arthritis rat model, possibly by inhibiting the toll-like receptor 2/myeloid differentiation factor 88/NF-kappa B p65 signaling pathway [34]. Geniposide exhibited anti-inflammatory and antiangiogenesis pharmacological effects through the inhibition of vascular endothelial growth factor-induced angiogenesis in vascular endothelial cells by reducing the translocation of sphingosine kinase 1 [35]. *Poria cocos* polysaccharide enhanced the secretion of immune stimulants but inhibited the secretion of immune inhibitors, enhancing the host immune response [36]. Icariin inhibited cell proliferation by interfering with the cell cycle in RA fibroblasts, including synovial cells, promoting mitochondria-dependent apoptosis and intracellular reactive oxygen species production, which potentially improves RA outcomes [37]. *Cinnamomum cassia* extract had a therapeutic effect on RA, which was attributed to its antiproliferation and antimigration effects on synovial fibroblasts [38]. A systematic review showed that curcumin had a significant effect on the clinical and inflammatory parameters of RA and significantly improved morning stiffness, walking time, and joint swelling [39]. Thus, the pharmacological effects of TCM support the use of TCMCP for reducing the risk of RA-related complications. These results demonstrated that TCMCPs could act as a protective factor against RA-related complications (readmission, Sjogren's syndrome, surgical treatment, and all-cause death).

However, we also found that RA patients with comorbidities such as hypertension or hyperlipidemia had a significantly high risk of readmission. A study showed that hypertension and dyslipidemia were the most common complications of RA [40]. Consistent with our results, these classic complications increased the risk of recurrence of RA inflammation [41], potentially contributing to increased readmission of RA patients. Advanced age and hypertension were shown to be significantly associated with the extra-articular manifestations of RA [42, 43], which is consistent with our findings. An analysis based on British electronic medical records showed that the incidence of joint replacement increased with age [12]. Our results revealed a 94.7% increased risk of surgical treatment in patients with RA aged 57 years and older, which corroborates findings from previous studies. Consistent with other studies, our results also showed that older patients with RA, men, and those with hypertension and diabetes had a higher risk of death [44–46]. Collectively, these results show that advanced age is a significant risk factor for extra-articular diseases, surgical treatment, and all-cause death. In addition, comorbidity hypertension is a risk factor for admission, extra-articular diseases, and all-cause death, whereas hyperlipidemia and diabetes are risk factors for recurrent admission and all-cause death, respectively. Among patients with RA, the risk of all-cause death is higher in men than in women.

Our study further found that medium- and high-exposure intensity, especially high-exposure intensity, were significantly associated with a reduced risk of RA-related complications. This indicates that long-term treatment with TCM could decrease the frequency of RA-related complications, which is consistent with the results of previous clinical data mining studies [16, 47]. Our results also suggest that long-term exposure to Xin'an Jianpi Tongbi preparation reduces RA-related complications.

This study had some notable limitations. First, there were no radiological data in our research to measure the severity of RA disease. Although, early on, we retrieved radiological data from the hospital information system, these data were textual, and we lacked models and algorithms to process textual data. Second, biologic DMARDs were not included in this study owing to insufficient data, which constitutes a major difference from the common practice in RA treatment and prevents appropriate comparisons with most of the literature on RA. Third, the recurrence frequency per unit time was not calculated for the frequently hospitalized patients, which differs from the common practice for RA treatment and also hinders proper comparison with most of the literature on RA. Fourth, the lack of data on adverse events of TCMCPs in this study did not allow a comprehensive analysis of the role of the drugs. Finally, we only studied Sjogren's syndrome and lacked data on other extra-articular manifestations of RA, which makes our findings one-sided. We intend to address these limitations in our future research. Nevertheless, our study has two significant strengths: the clinical advantage of using TCM and the statistical advantage of using large samples. This was a population-based cohort study, which included the clinical administration of medication to a population, making our results more clinically acceptable. The large sample size provides sufficient statistical ability to study the improvement effect of TCMCP on RA-related clinical indicators.

## 5. Conclusion

This population-based cohort study showed that TCMCP use, as well as long-term exposure to TCMCP in patients with RA, decreased the risk of RA-related complications, including readmission, Sjogren's syndrome, surgical treatment, and all-cause death. These findings are expected to inform clinical decisions regarding the use of TCMCP in RA management.

## Figures and Tables

**Figure 1:**
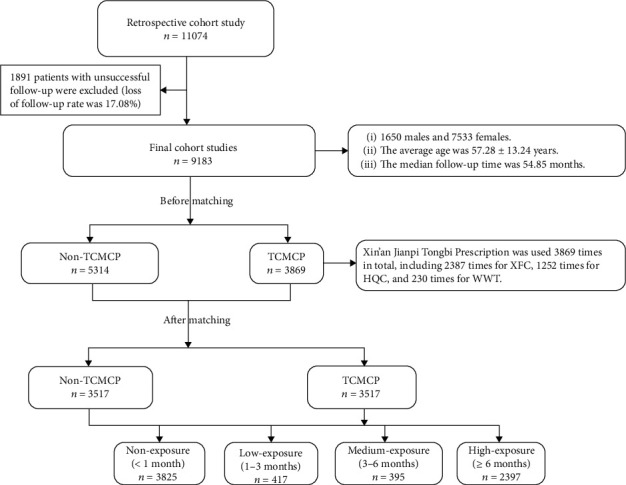
Flow chart of inclusion in the cohort. TCMCP: users of traditional Chinese medicine compound preparation; non-TCMCP: nonusers of traditional Chinese medicine compound preparation.

**Figure 2:**
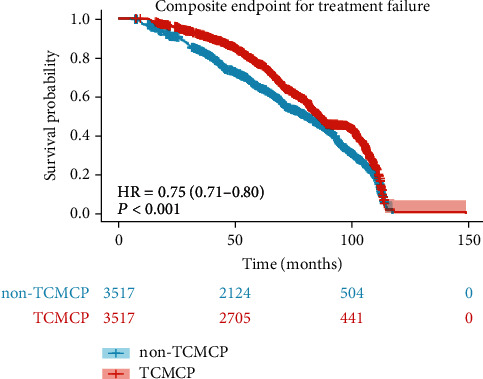
Kaplan-Meier curves for a composite endpoint for treatment failure for TCMCP users versus non-TCMCP users. The composite endpoint prognosis for treatment failure was better in TCMCP users (HR = 0.75 (0.71-0.80), *p* < 0.001) than in non-TCMCP users. The *p* value represents the comparison in composite endpoint for treatment failure predicted by log-rank test between TCMCP users and non-TCMCP users. TCMCP: traditional Chinese medicine compound preparation.

**Figure 3:**
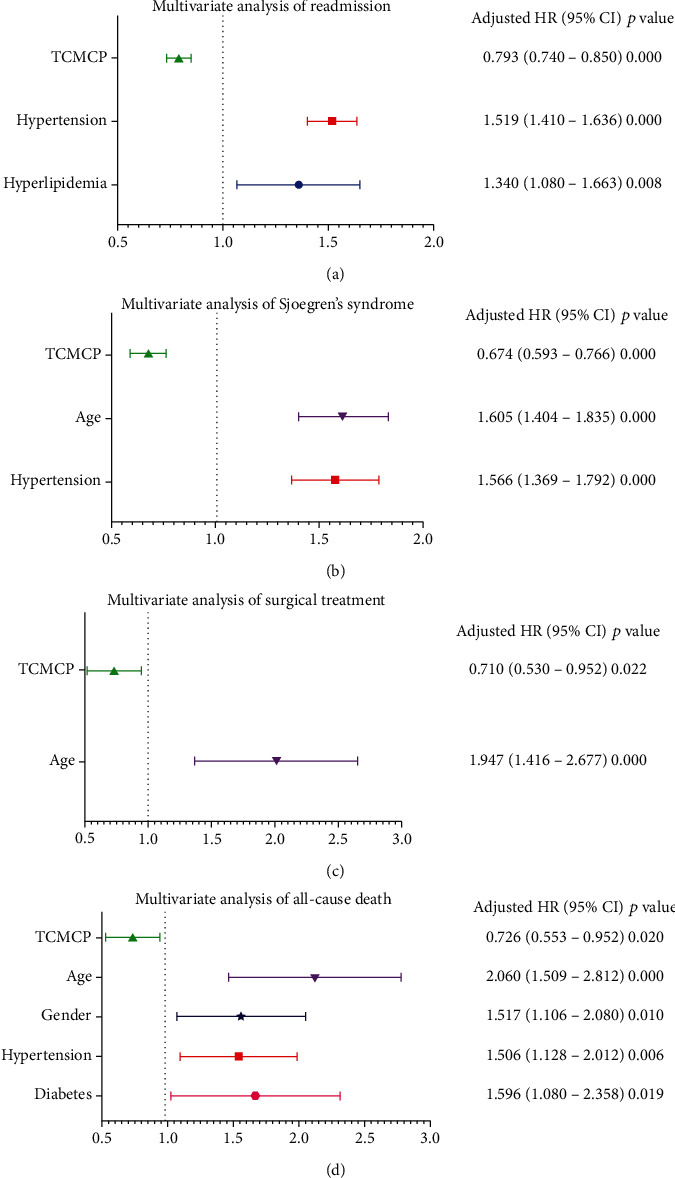
Multivariate regression analysis of rheumatoid arthritis-related complications: (a) TCMCP is a protective factor of recurrent admission, whereas hypertension and hyperlipidemia are risk factors; (b) TCMCP is a protective factor of Sjogren's syndrome, whereas higher age and hypertension are risk factors; (c) TCMCP is a protective factor of surgical treatment whereas higher age is a risk factor; (d) TCMCP is a protective factor of all-cause death, whereas higher age, male, hypertension, and diabetes are risk factors. TCMCP: traditional Chinese medicine compound preparation.

**Table 1:** Baseline characteristics of TCMCP and non-TCMCP patients, matched and unmatched by propensity score.

	Before matching					After matching				
	Total (*n* = 9183)	TCMCP (*n* = 3869)	Non-TCMCP (*n* = 5314)	*χ* ^2^	*p* value	Total (*n* = 7034)	TCMCP (*n* = 3517)	Non-TCMCP (*n* = 3517)	*χ* ^2^	*p* value
Age (year)				10.649	0.001				3.395	0.065
<57	4643 (50.6%)	1879 (48.6%)	2764 (52.0%)			3223 (45.8%)	1650 (46.9%)	1573 (44.7%)		
≥57	4540 (49.4%)	1990 (51.4%)	2550 (48.0%)			3811 (54.2%)	1867 (53.1%)	1944 (55.3%)		
Sex				55.078	<0.001				1.164	0.281
Female	7534 (82.0%)	3039 (78.5%)	4494 (84.6%)			5923 (84.2%)	2945 (83.7%)	2978 (84.7%)		
Male	1650 (18.0%)	830 (21.5%)	820 (15.4%)			1111 (15.8%)	572 (16.3%)	539 (15.3%)		
Underlying diseases										
Hypertension	2804 (30.5%)	988 (25.5%)	1816 (34.2%)	78.751	<0.001	2111 (30.0%)	1072 (30.5%)	1039 (29.5%)	0.737	0.391
Diabetes	903 (9.8%)	422 (10.9%)	481 (12.4%)	8.695	0.003	647 (9.20%)	315 (8.96%)	332 (9.44%)	0.492	0.483
Hyperlipidemia	250 (2.7%)	99 (2.6%)	151 (2.8%)	0.676	0.411	179 (2.54%)	85 (2.42%)	94 (2.67%)	0.464	0.496
Treatment										
cDMARDs	6276 (68.3%)	2697 (69.7%)	3579 (67.4%)	5.752	0.016	4968 (70.6%)	2507 (71.3%)	2461 (70.0%)	1.450	0.229
Corticosteroid	5597 (60.9%)	2191 (56.6%)	3406 (64.1%)	52.423	<0.001	4291 (61.0%)	2117 (60.2%)	2174 (61.8%)	1.942	0.163

Note: TCMCP: users of traditional Chinese medicine compound preparation; non-TCMCP: nonusers of traditional Chinese medicine compound preparation.

**Table 2:** Changes in the rheumatoid arthritis-related inflammatory and immune indices after administration of TCMCP (*n* = 3517).

	Before treatment	After treatment	*Z*	*p* value	Reference ranges
ESR (median (Q1, Q3), mm/h)	48.0 (29.00, 70.00)	28.00 (16.00, 44.00)	-34.498	<0.001	2-6
CRP (median (Q1, Q3), mg/L)	24.29 (7.00, 50.57)	2.00 (0.48, 8.66)	-40.240	<0.001	0-5
IgA (median (Q1, Q3), g/L)	2.65 (2.00, 3.48)	2.48 (1.91, 3.22)	-15.313	<0.001	0.7-4.06
IgG (median (Q1, Q3), g/L)	12.98 (10.07, 16.70)	12.00 (9.56, 15.30)	-18.241	<0.001	6.8-14.5
IgM (median (Q1, Q3), g/L)	1.26 (0.89, 1.66)	1.28 (0.91, 1.72)	-3.983	<0.001	0.3-2.2
C3 (median (Q1, Q3), g/L)	110.60 (87.70, 129.90)	100.20 (81.70, 115.10)	-23.671	<0.001	75-135
C4 (median (Q1, Q3), g/L)	23.50 (15.6, 30.10)	19.30 (12.60, 24.90)	-27.145	<0.001	9-36
Anti-CCP (median (Q1, Q3), mmol/L)	240.90 (105.92, 473.98)	220.45 (84.15, 452.69)	-6.170	<0.001	<25
RF (median (Q1, Q3), U/mL)	101.70 (33.55, 244.50)	88.85 (27.53, 216.45)	-20.372	<0.001	0-14

Note: TCMCP: traditional Chinese medicine compound preparation; ESR: erythrocyte sedimentation rate; CRP: C-reactive protein; IgA: immunoglobulin A; IgM: immunoglobulin M; IgG: immunoglobulin G; C3: complement C3; C4: complement C4; anti-CCP: anti-cyclic citrullinated peptide; RF: rheumatoid factor. *Z* is the standardized test statistics before and after treatment of TCMCP. The *p* value is compared before and after treatment with TCMCP.

**Table 3:** Association between traditional Chinese medicine compound preparation with rheumatoid arthritis-related inflammatory and immune indices.

TCMCP	Indexes	*χ* ^2^	*p* value	OR	95% CI
XFC	CRP ↓	4.264	0.039	1.216	1.010-1.463
XFC	ESR ↓	9.026	0.003	1.298	1.095-1.539
XFC	C4 ↓	4.820	0.028	1.258	1.025-1.544
HQC	CRP ↓	24.65	<0.001	1.641	1.348-1.998
HQC	ESR ↓	10.001	0.002	1.324	1.112-1.575
HQC	C4 ↓	5.095	0.024	1.272	1.032-1.569
HQC	IgG ↓	5.509	0.019	1.247	1.037-1.499
HQC	IgA ↓	5.285	0.022	1.237	1.032-1.484

Note: TCMCP: users of traditional Chinese medicine compound preparation; XFC: Xinfeng capsule; HQC: Huangqin Qingre capsule; ESR: erythrocyte sedimentation rate; CRP: C-reactive protein; C4: complement C4; IgG: immunoglobulin G; IgA: immunoglobulin A. “↓” represents the decrease of quantitative variables, indicating that the laboratory indicators improved after TCMCP treatment.

**Table 4:** Analysis of risk factors for the four rheumatoid arthritis-related complications using the COX regression model.

	Number of endpoint events	Univariate analysis			Multivariate analysis		
		HR	95% CI	*p* value	HR	95% CI	*p* value
*Readmission*	3253 (46.2%)						
TCMCP	1510 (21.5%)	0.786	0.733-0.842	<0.001	0.793	0.740-0.850	<0.001
Age (year)		1.041	0.971-1.115	0.257			
<57	1459 (20.7%)						
≥57	1794 (25.5%)						
Sex		0.910	0.826-1.002	0.054			
Female	2767 (39.3%)						
Male	486 (6.9%)						
Underlying diseases							
Hypertension	1043 (14.8%)	1.525	1.416-1.643	<0.001	1.519	1.410-1.636	<0.001
Diabetes	277 (3.9%)	1.091	0.965-1.234	0.165			
Hyperlipidemia	85 (1.2%)	1.350	1.088-1.675	0.006	1.340	1.080-1.663	0.008
*Sjogren's syndrome*	965 (13.7%)						
TCMCP	414 (5.9%)	0.684	0.602-0.777	<0.001	0.674	0.593-0.766	<0.001
Age (year)		1.633	1.429-1.865	<0.001	1.605	1.404-1.835	<0.001
<57	331 (4.7%)						
≥57	634 (9.0%)						
Sex		1.123	0.952-1.325	0.167			
Female	793 (11.3%)						
Male	172 (2.4%)						
Underlying diseases							
Hypertension	335 (4.8%)	1.660	1.451-1.898	<0.001	1.566	1.369-1.792	<0.001
Diabetes	88 (1.3%)	1.163	0.934-1.448	0.177			
Hyperlipidemia	27 (0.4%)	1.402	0.956-2.057	0.084			
*Surgical treatment*	182 (2.6%)						
TCMCP	82 (1.2%)	0.724	0.540-0.970	0.030	0.710	0.530-0.952	0.022
Age (year)		1.990	1.452-2.726	<0.001	1.947	1.416-2.677	<0.001
<57	56 (0.8%)						
≥57	126 (1.8%)						
Sex		1.137	0.780-1.388	0.505			
Female	149 (2.1%)						
Male	33 (0.5%)						
Underlying diseases							
Hypertension	52 (0.7%)	1.005	0.728-1.388	0.975			
Diabetes	23 (0.3%)	1.581	1.021-2.448	0.040	1.366	0.878-2.126	0.167
Hyperlipidemia	8 (0.1%)	1.905	0.938-3.871	0.075			
*All-cause death*	215 (3.1%)						
TCMCP	93 (1.3%)	0.758	0.578-0.994	0.045	0.726	0.553-0.952	0.020
Age (year)		2.314	1.710-3.134	<0.001	2.060	1.509-2.812	<0.001
<57	57 (0.8%)						
≥57	158 (2.2%)						
Sex		1.687	1.237-2.301	0.001	1.517	1.106-2.080	0.010
Female	162 (2.3%)						
Male	53 (0.8%)						
Underlying diseases							
Hypertension	72 (1.0%)	1.590	1.192-2.119	0.002	1.506	1.128-2.012	0.006
Diabetes	30 (0.4%)	1.880	1.278-2.766	0.001	1.596	1.080-2.358	0.019
Hyperlipidemia	5 (0.1%)	1.209	0.497-2.938	0.676			

Abbreviation: TCMCP: traditional Chinese medicine compound preparation.

**Table 5:** Hazard ratios and 95% confidence intervals of the risk of rheumatoid arthritis-related complications at different exposure times.

Exposure group	Total	Number of complications	HR	95% CI	*p* value
None (<1 month)	3825	2877 (75.2%)	1		
Low (1-3 months)	417	271 (65.0%)	0.994	0.868-1.137	0.926
Medium (3-6 months)	395	241 (61.0%)	0.796	0.691-0.918	0.002
High (≥6 months)	2397	1226 (51.1%)	0.699	0.650-0.751	<0.001

## Data Availability

All relevant data are included in the manuscript.

## References

[1] Maden M. (2020). RA signaling in limb development and regeneration in different species. *Sub-Cellular Biochemistry*.

[2] Giannini D., Antonucci M., Petrelli F., Bilia S., Alunno A., Puxeddu I. (2020). One year in review 2020: pathogenesis of rheumatoid arthritis. *Clinical and Experimental Rheumatology*.

[3] van der Woude D., van der Helm-van Mil A. H. M. (2018). Update on the epidemiology, risk factors, and disease outcomes of rheumatoid arthritis. *Best Practice & Research. Clinical Rheumatology*.

[4] Huang J., Fu X., Chen X., Li Z., Huang Y., Liang C. (2021). Promising therapeutic targets for treatment of rheumatoid arthritis. *Frontiers in Immunology*.

[5] Liu Z. C., Gao L., Zhang W. H., Wang J., Liu R. R., Cao B. H. (2020). Effects of a 4-week Omaha System transitional care programme on rheumatoid arthritis patients' self-efficacy, health status, and readmission in mainland China: a randomized controlled trial. *International Journal of Nursing Practice*.

[6] Figus F. A., Piga M., Azzolin I., McConnell R., Iagnocco A. (2021). Rheumatoid arthritis: extra-articular manifestations and comorbidities. *Autoimmunity Reviews*.

[7] England B. R., Thiele G. M., Anderson D. R., Mikuls T. R. (2018). Increased cardiovascular risk in rheumatoid arthritis: mechanisms and implications. *BMJ*.

[8] Wasserman A. (2018). Rheumatoid arthritis: common questions about diagnosis and management. *American Family Physician*.

[9] Brzustewicz E., Henc I., Daca A. (2017). Autoantibodies, C-reactive protein, erythrocyte sedimentation rate and serum cytokine profiling in monitoring of early treatment. *Central European Journal of Immunology*.

[10] Pirmardvand Chegini S., Varshosaz J., Taymouri S. (2018). Recent approaches for targeted drug delivery in rheumatoid arthritis diagnosis and treatment. *Artificial Cells, Nanomedicine, and Biotechnology*.

[11] Lin Y. J., Anzaghe M., Schülke S. (2020). Update on the pathomechanism, diagnosis, and treatment options for rheumatoid arthritis. *Cell*.

[12] Hawley S., Edwards C. J., Arden N. K. (2020). Descriptive epidemiology of hip and knee replacement in rheumatoid arthritis: an analysis of UK electronic medical records. *Seminars in Arthritis and Rheumatism*.

[13] Turkiewicz A., Neogi T., Björk J., Peat G., Englund M. (2016). All-cause mortality in knee and hip osteoarthritis and rheumatoid arthritis. *Epidemiology*.

[14] Kerola A. M., Kazemi A., Rollefstad S. (2022). All-cause and cause-specific mortality in rheumatoid arthritis, psoriatic arthritis and axial spondyloarthritis: a nationwide registry study. *Rheumatology*.

[15] Littlejohn E. A., Monrad S. U. (2018). Early diagnosis and treatment of rheumatoid arthritis. *Primary Care*.

[16] Fang Y., Liu J., Xin L. (2020). Identifying compound effect of drugs on rheumatoid arthritis treatment based on the association rule and a random walking-based model. *BioMed Research International*.

[17] Lv Q. W., Zhang W., Shi Q. (2015). Comparison of *Tripterygium wilfordii* Hook F with methotrexate in the treatment of active rheumatoid arthritis (TRIFRA): a randomised, controlled clinical trial. *Annals of the Rheumatic Diseases*.

[18] Liu J., Huang C. B., Wang Y. (2013). Chinese herbal medicine Xinfeng Capsule in treatment of rheumatoid arthritis: study protocol of a multicenter randomized controlled trial. *Journal of Integrative Medicine*.

[19] Li S., Wan L., Zhao L. (2022). Clinical observation of Huangqin Qingre Chubi capsule in treating rheumatoid arthritis and its effect on serum M1 and M2 inflammatory factors. *Zhong Yao Yao Li Yu Lin Chuang Za Zhi*.

[20] Zhang Y., Liu J., Jiang H. (2020). Study on the effect of Wuwei Wentong Chubi Capsule on rheumatoid arthritis patients with cold-dampness syndrome based on association rules. *Feng Shi Bing Yu Guan Jie Yan Za Zhi*.

[21] Liu J., Wang Y., Huang C. (2015). Efficacy and safety of Xinfeng capsule in patients with rheumatoid arthritis: a multi-center parallel-group double-blind randomized controlled trial. *Journal of Traditional Chinese Medicine*.

[22] Meng M., Wu X., Wang X. Y., Liu J., Du D., Ge Z. (2012). Study on the quality evaluation index method of Xinfeng Capsules. *Clinical Journal of Traditional Chinese Medicine*.

[23] Wang X., Chang J., Zhou G. (2021). The traditional Chinese medicine compound Huangqin Qingre Chubi capsule inhibits the pathogenesis of rheumatoid arthritis through the CUL4B/Wnt pathway. *Frontiers in Pharmacology*.

[24] Liu J. Q., Liu X. C., Liu J., Zhang Y. Y., Wang T. J., Zhou A. (2022). Preliminary study on HPLC fingerprint of Huangqin Qingre Chubi Capsules and determination of three components. *Chinese Medicinal Biotechnology*.

[25] Jiang H., Liu J., Wang Y. (2021). Screening the Q-markers of TCMs from RA rat plasma using UHPLC-QTOF/MS technique for the comprehensive evaluation of Wu-Wei-Wen-Tong Capsule. *Journal of Mass Spectrometry*.

[26] Arnett F. C., Edworthy S. M., Bloch D. A. (1988). The American Rheumatism Association 1987 revised criteria for the classification of rheumatoid arthritis. *Arthritis and Rheumatism*.

[27] Jiang H., Wu F. R., Liu J., Qin X. J., Jiang N. N., Li W. P. (2019). Effect of astragalosides on long non-coding RNA expression profiles in rats with adjuvant-induced arthritis. *International Journal of Molecular Medicine*.

[28] Liu X. Y., Xu L., Wang Y. (2017). Protective effects of total flavonoids of *Astragalus* against adjuvant- induced arthritis in rats by regulating OPG/RANKL/NF- *κ*B pathway. *International Immunopharmacology*.

[29] Zhang C., Zhang W., Shi R., Tang B., Xie S. (2019). Coix lachryma-jobi extract ameliorates inflammation and oxidative stress in a complete Freund's adjuvant-induced rheumatoid arthritis model. *Pharmaceutical Biology*.

[30] Wang J., Liu J., Wen J., Wang X. (2022). Triptolide inhibits inflammatory response and migration of fibroblast like synovial cells in rheumatoid arthritis through the circRNA 0003353/JAK2/STAT3 signaling pathway. *Nan Fang Yi Ke Da Xue Xue Bao*.

[31] Lin N., Zhang Y. Q., Jiang Q. (2021). Clinical practice guideline for *Tripterygium* glycosides/*Tripterygium wilfordii* tablets in the treatment of rheumatoid arthritis. *Frontiers in Pharmacology*.

[32] Wen J. T., Liu J., Wan L. (2022). Triptolide inhibits cell growth and inflammatory response of fibroblast-like synoviocytes by modulating hsa-circ-0003353/microRNA-31-5p/CDK1 axis in rheumatoid arthritis. *International Immunopharmacology*.

[33] Zhong H., Zhao J. (2003). Clinical application of insect drugs. *Journal of Traditional Chinese Medicine*.

[34] Bai L., Bai Y., Yang Y. (2020). Baicalin alleviates collagen-induced arthritis and suppresses TLR2/MYD88/NF-*κ*B p65 signaling in rats and HFLS-RAs. *Molecular Medicine Reports*.

[35] Wang Y., Wu H., Gui B. J. (2022). Geniposide alleviates VEGF-induced angiogenesis by inhibiting VEGFR2/PKC/ERK1/2-mediated SphK1 translocation. *Phytomedicine*.

[36] Ríos J. L. (2011). Chemical constituents and pharmacological properties of Poria cocos. *Planta Medica*.

[37] Pu L., Meng Q., Li S., Liu B., Li F. (2021). Icariin arrests cell cycle progression and induces cell apoptosis through the mitochondrial pathway in human fibroblast-like synoviocytes. *European Journal of Pharmacology*.

[38] Liu J., Zhang Q., Li R. L. (2020). Anti-proliferation and anti-migration effects of an aqueous extract of *Cinnamomi ramulus* on MH7A rheumatoid arthritis-derived fibroblast-like synoviocytes through induction of apoptosis, cell arrest and suppression of matrix metalloproteinase. *Pharmaceutical Biology*.

[39] Pourhabibi-Zarandi F., Shojaei-Zarghani S., Rafraf M. (2021). Curcumin and rheumatoid arthritis: a systematic review of literature. *International Journal of Clinical Practice*.

[40] Namas R., Joshi A., Ali Z., Al Saleh J., Abuzakouk M. (2019). Demographic and clinical patterns of rheumatoid arthritis in an Emirati cohort from United Arab Emirates. *International Journal of Rheumatology*.

[41] Fragoulis G. E., Panayotidis I., Nikiphorou E. (2020). Cardiovascular risk in rheumatoid arthritis and mechanistic links: from pathophysiology to treatment. *Current Vascular Pharmacology*.

[42] Chen X., Zhang M., Wang T., Li Y., Wei M. (2020). Influence factors of extra-articular manifestations in rheumatoid arthritis. *Open Medicine*.

[43] Ortega-Hernandez O. D., Pineda-Tamayo R., Pardo A. L., Rojas-Villarraga A., Anaya J. M. (2009). Cardiovascular disease is associated with extra-articular manifestations in patients with rheumatoid arthritis. *Clinical Rheumatology*.

[44] Kim S. U., Kim B. K., Park J. Y. (2020). Fibrosis-4 index at diagnosis can predict all-cause mortality in patients with rheumatoid arthritis: a retrospective monocentric study. *Modern Rheumatology*.

[45] Lee E. E., Shin A., Lee J. (2022). All-cause and cause-specific mortality of patients with rheumatoid arthritis in Korea: a nation-wide population-based study. *Joint, Bone, Spine*.

[46] Kuo C. F., Luo S. F., See L. C., Chou I. J., Chang H. C., Yu K. H. (2013). Rheumatoid arthritis prevalence, incidence, and mortality rates: a nationwide population study in Taiwan. *Rheumatology International*.

[47] Zhou Q., Liu J., Xin L. (2021). Exploratory compatibility regularity of traditional Chinese medicine on osteoarthritis treatment: a data mining and random walk-based identification. *Evidence-based Complementary and Alternative Medicine*.

